# Metformin-associated lactic acidosis in an intensive care unit

**DOI:** 10.1186/cc7137

**Published:** 2008-11-26

**Authors:** Nicolas Peters, Nicolas Jay, Damien Barraud, Aurélie Cravoisy, Lionel Nace, Pierre-Edouard Bollaert, Sébastien Gibot

**Affiliations:** 1 Service de Néphrologie, CHU Brabois; Vandoeuvre les Nancy, 54500, France; 2 Laboratoire SPIEAO, Faculté de Médecine; Nancy Université, Nancy, 54000, France; 3 Service de Réanimation Médicale, Hôpital Central; Nancy, 54000, France

## Abstract

**Introduction:**

Metformin-associated lactic acidosis (MALA) is a classic side effect of metformin and is known to be a severe disease with a high mortality rate. The treatment of MALA with dialysis is controversial and is the subject of many case reports in the literature. We aimed to assess the prevalence of MALA in a 16-bed, university-affiliated, intensive care unit (ICU), and the effect of dialysis on patient outcome.

**Methods:**

Over a five-year period, we retrospectively identified all patients who were either admitted to the ICU with metformin as a usual medication, or who attempted suicide by metformin ingestion. Within this population, we selected patients presenting with lactic acidosis, thus defining MALA, and described their clinical and biological features.

**Results:**

MALA accounted for 0.84% of all admissions during the study period (30 MALA admissions over five years) and was associated with a 30% mortality rate. The only factors associated with a fatal outcome were the reason for admission in the ICU and the initial prothrombin time. Although patients who went on to haemodialysis had higher illness severity scores, as compared with those who were not dialysed, the mortality rates were similar between the two groups (31.3% versus 28.6%).

**Conclusions:**

MALA can be encountered in the ICU several times a year and still remains a life-threatening condition. Treatment is restricted mostly to supportive measures, although haemodialysis may possess a protective effect.

## Introduction

Since the UK Prospective Diabetes Study was published in 1998, metformin has become the standard of care for overweigh patients with diabetes [1]. Indeed, metformin has been shown to reduce the rate of cardiovascular disease within this population [1].

Metformin is a small molecule (165 kDa) with a 50% oral bioavailability; it does not undergo hepatic metabolism and the main route of elimination is renal tubular secretion. Metformin is not bound to proteins and its apparent volume of distribution is usually reported to be higher than 3 L/kg (63 to 646 L in total) attesting to the predominance of the intracellular location. Considering these data, metformin can theoretically be extracted from blood by haemodialysis if dialysis is conducted for long enough to mobilise the intracellular form.

Metformin-associated lactic acidosis (MALA) is a rare but classic side effect of metformin [2]. Two years after the introduction of this drug to the US market, a study showed an incidence of MALA of two to nine cases per 100,000 patients treated with metformin each year [3] with an associated mortality rate as high as 50%.

The physiopathology of MALA is complex and mostly unclear. However, this side effect seems to be closely related to the anti-hyperglycaemic effect of metformin [4]. It is also known that metformin impairs lactate clearance of the liver through the inhibition of complex I of the mitochondrial respiratory chain [5,6]. Although increased lactic acid production may be induced by haemodynamic instability and/or tissue hypoxia associated with severe metformin overdose or any underlying unstable cardiovascular or respiratory condition, lactic acidosis is predominantely due to a lack of lactate's clearance than to an increased production.

Intensivists may be confronted with MALA because of its potential severity. Nevertheless, the treatment of MALA is mostly restricted to supportive measures as there is no specific therapeutics. Haemodialysis is appealing as it can buffer acidosis and theoretically extract metformin from blood [7,8]. Unfortunately, this technique has not gained widespread acceptance due to the lack of well-conducted studies. Indeed, only case reports have dealt with this subject [9-12].

We aimed to assess the prevalence of MALA in a 16-bed, university-affiliated, intensive care unit (ICU), and the effect of dialysis on patient outcome.

## Materials and methods

### Study design and definitions

The study was conducted at the Hopital Central, University of Nancy, France. The hospital records of all patients admitted to the ICU between August 2002 and August 2007 were retrospectively evaluated and patients were included if they met the following criteria: current metformin medication as their usual treatment; metformin overdose in the setting of a suicide attempt; and lactic acidosis defined by lactate concentration higher than 5 mmol/L and bicarbonate level less than 22 mmol/L. Patients were not enrolled if a limitation of care was decided on admission.

The retrospective and non-interventional nature of this study waived the need for ethics committee approval.

Clinical and laboratory features at admission and during the ICU stay were studied: simplified acute physiology score (SAPS) II of severity, Charlson index (used to assess the heaviness of comorbidities) [13], age, sex, reason for admission to the ICU, blood pressure, respiratory rate and vasopressor requirement. Acute renal failure was defined according to the RIFLE (acronym indicating Risk of renal dysfunction; Injury to the kidney; Failure of kidney function, Loss of kidney function and End-stage kidney disease) criteria (increase creatinine times three or glomerular filtration rate decrease of more than 75%; urine output less than 0.3 mL/kg/hour every 24 hours or anuria for longer than 12 hours despite appropriate fluid replacement) [14]. Biological data recorded were arterial pH, blood lactate, bicarbonate, glucose and creatinine concentrations, as well as prothrombin time.

### Patient population

The patients were divided according to their 28-day outcome in order to investigate if there were differences in relation to all the studied parameters. The population was also split regarding the use of haemodialysis. Due to the retrospective design, no rules precluded the use of haemodialysis.

### Statistical analysis

Results were expressed as mean ± standard deviation or median (range) for quantitative variables. Comparisons between groups were performed with a Student's t-test, Fisher's exact test or Mann-Whitney test when appropriate. Analysis of associations between death and categorised risk factors was done with Fisher's exact test and Pearson's chi-square test. Statistical analyses were conducted using the R software [15] and a two-tailed p < 0.05 was deemed significant.

## Results

**Figure 1:**
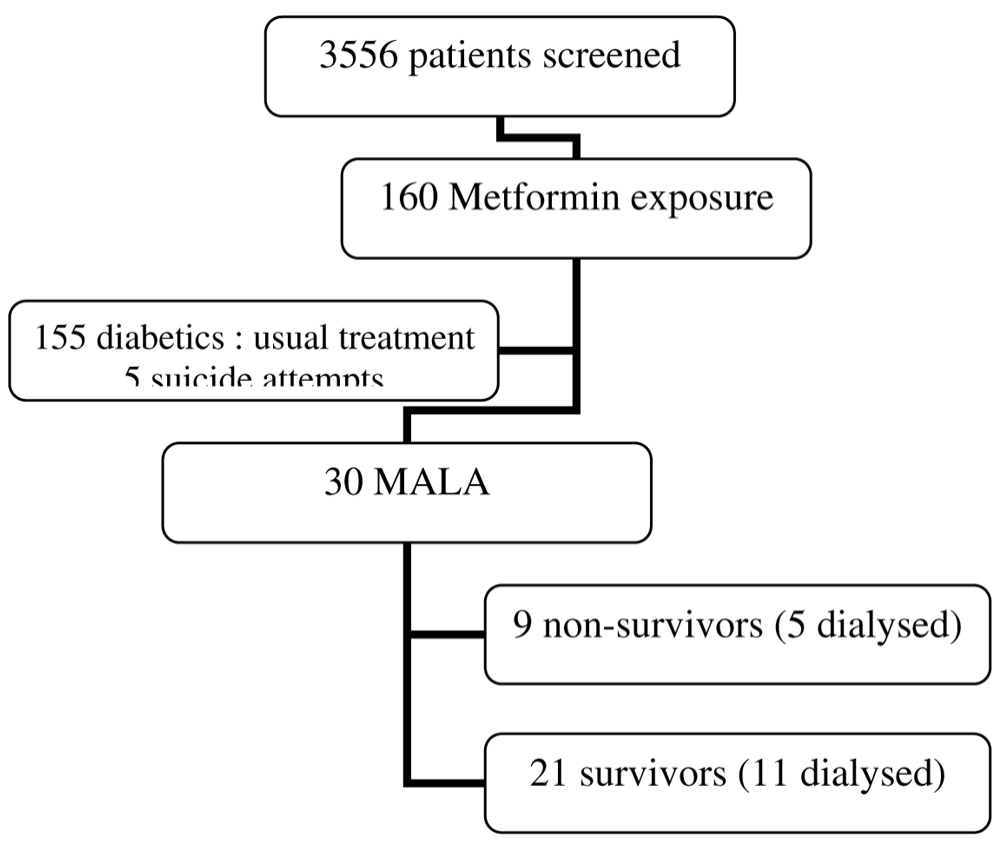
**Flow chart of patient outcome**. MALA = metformin-associated lactic acidosis.

During the study period, 3556 patients were admitted into the ICU. Among this group, 160 were identified as having been exposed to metformin but only 30 (18.7%) presented with MALA (Figure 1). Reasons for ICU admission were shock (n = 12), acute renal failure (n = 9), acute respiratory distress syndrome or acute lung injury (n = 3), suicide attempt (n = 3), cardiac arrest (n = 2) and intracerebral haemorrhage (n = 1). Thus, no patient was referred to the ICU because of MALA but with an acute disorder associated with the development of MALA.

Patients' characteristics are reported in Table 1. Length of stay in the ICU was 12.8 ± 17.7 days and the 28-day mortality rate was 30%.

**Table 1:** Patients' characteristics on admission according to their outcome.

	All patients (n = 30)	Survivors (n = 21)	Non-survivors (n = 9)	p value
				

Age (years)	66.8 ± 13.6	66.1 ± 12.6	68.4 ± 11.4	0.90

Sex ratio male/female	15/15	7/14	8/1	0.10

SAPSII	60 ± 21	53 ± 12	75 ± 23	0.004

Charlson index	3 ± 2	3 ± 1	3 ± 2	0.28

Reason for ICU referral				

Shock (%)	40.0	23.8	77.8	0.002

Acute renal failure (%)	30.0	42.9	0	0.03

Suicide attempt (%)	10.0	14.3	0	0.31

Other (%)	20.0	19.0	22.2	0.86

				

Mechanical ventilation (%)	36.7	33.3	55.6	0.68

Vasopressors (%)	60	52.4	77.8	0.24

Acute renal failure (%)	80	71.4	100	0.14

				

Arterial pH	7.18 ± 0.19	7.19 ± 0.21	7.16 ± 0.10	0.15

Arterial lactate (mmol/L)	9.9 ± 4.1	10.2 ± 4.3	9.2 ± 3.7	0.58

Arterial bicarbonate (mmol/L)	13.2 ± 5.7	11.8 ± 6.1	16.3 ± 3.0	0.12

Creatinine (mg/L)	45.0 ± 30.7	50.9 ± 38.6	31.5 ± 12.5	0.30

Prothrombin time (sec)	19 ± 2	17 ± 2	21 ± 3	0.04

				

Length of stay in ICU (days)	12.8 ± 17.7	8.1 ± 10.2	23.7 ± 22.8	0.35

ICU = intensive care unit; SAPSII: simplified acute physiology score II.

When compared with survivors, non-survivors were more often referred to the ICU for shock (p = 0.002), displayed a higher SAPSII score (p = 0.004) and a higher prothrombin time (p = 0.04). The degree of lactic acidosis did not differ between groups, nor did the requirement for mechanical ventilation, vasopressors or dialysis.

On admission, 80% of our patients presented with acute renal failure, of whom 62.5% underwent dialysis therapy (no patient had a history of chronic renal failure). Only one patient with an unaltered renal function underwent dialysis therapy because of severe acidosis. Of note, 55.6% of survivors were dialysed as compared with 52.4% of non-survivors (p = 0.8). Intermittent veno-venous haemodialysis with the use of a bicarbonate buffer was performed and we observed no dialysis disequilibrium syndromes.

We also compared patients who underwent dialysis and patients who did not (Table 2). There was a trend for a higher severity among dialysed patients as reflected by a higher SAPSII score (p = 0.04), and trend towards a more frequent requirement for supportive therapies (vasopressors, mechanical ventilation; not statistically significant) and a higher degree of metabolic acidosis. Despite this higher severity, the mortality rate did not differ between dialysed and non-dialysed patients.

**Table 2:** Patients' characteristics according to their dialysis status.

	Dialysis (n = 16)	No dialysis (n = 14)	P value
			

Age (years)	66.9 ± 11.6	66.6 ± 12.4	0.57

Sex ratio male/female	7/9	7/7	0.61

SAPSII	61 ± 12	43 ± 11	0.04

Charlson index	3.5 ± 1.0	2.5 ± 0.7	0.12

Reason for ICU referral			

Shock (%)	43.8	35.7	0.58

Acute renal failure (%)	37.5	21.4	0.11

Suicide attempt (%)	12.5	7.1	0.37

Other (%)	6.3	35.8	0.01

			

Mechanical ventilation (%)	43.8	28.6	0.16

Vasopressors (%)	68.8	50	0.13

Acute renal failure (%)	93.8	64.3	0.02

			

Arterial pH	7.11 ± 0.20	7.26 ± 0.12	0.07

Arterial lactate (mmol/L)	11.2 ± 4.8	8.4 ± 2.6	0.24

Arterial bicarbonate (mmol/L)	9.8 ± 5.8	16.4 ± 3.2	0.006

Creatinine (mg/L)	60.4 ± 38.0	27.6 ± 16.6	0.01

			

Length of stay in ICU (days)	19.4 ± 8.2	5.3 ± 2.1	0.009

Mortality rate (%)	31.3	28.6	0.82

ICU = intensive care unit; SAPSII: simplified acute physiology score II.

## Discussion

The definition and diagnostic criteria of MALA are based on metformin exposure associated with the presence of lactic acidosis. We therefore enrolled patients with a lactate concentration of 5 mmol/L or higher and a bicarbonate level of less than 22 mmol/L evidenced before or at admission into the ICU. Routine assessment of metformin plasma concentration is not easy and of no value because metformin is essentially an intracellular toxin. Moreover, as any concentration of metformin may impair liver lactate clearance, it is worth considering that the observation of lactic acidosis concomitant to a recent ingestion of metformin may, at least in part, be related to this drug. We then choose to consider MALA as lactic acidosis observed in all patients with a recent ingestion of metformin.

The current study describes 30 cases of MALA and there is, to the best of our knowledge, only one study reporting a larger series of cases but not focussed on critically ill patients [3]. We found that MALA was present in about 1% of patients admitted to the ICU, and indeed metformin is a factor that is detrimental to the outcome in the setting of an acute disease rather than the primary reason for referral to the ICU.

The 30% death rate we observed is lower than previously reported [3]. This may be because of a better awareness of this side effect, as well as a continuous improvement of care in the ICUs. We also noted a high rate (80%) of acute renal failure on admission; this highlights the role of metformin accumulation in the pathophysiology of MALA.

The leading factor associated with a fatal outcome is unsurprisingly the reason for ICU referral: admission for shock is associated with an increase risk of death as compared with referral for acute renal failure or suicide attempt. Interestingly, the degree of lactic acidosis was not associated with outcome. The fact that prothrombin time was related to survival may reflect the importance of liver function in the pathophysiology of MALA, but may also just be a consequence of shock.

We also observed a trend towards higher illness severity scores in the dialysed group of patients: SAPSII score was higher and needs for mechanical ventilation or vasopressors tended to be more frequent in dialysed patients. The fact that despite these higher illness severity scores the mortality rate was no different to that of the non-dialysed patients may suggest a beneficial affect of dialysis. Unfortunately, more detailed analyses with adjustment for severity were precluded by the small size of our population. Therefore, the protective effect of dialysis remains hypothetical.

Apart from haemodialysis, continuous veno-venous haemofiltration or haemodiafiltration, dichloroacetate and/or sodium bicarbonate infusions have been proposed as part of the treatment of MALA [8,16], but again only from small case series discussions.

Several limitations of this study must be acknowledged. First, the small size of our series did not allow us to make multiple comparisons and multivariate analyses. Unfortunately, there is no larger study dealing with MALA in the ICU. Second, the current study was retrospective and therefore we couldn't correct the bias by which only the more severely ill patients were dialysed. Finally, in some situations (e.g. cardiac arrest or shock), the exact role of metformin in explaining the degree of lactic acidosis could not be definitely ascertained as these conditions may *per se *be associated with hyperlactataemia. Nevertheless, we strictly applied the recommended definition of MALA for the inclusion of our patients considering that even in these above mentioned conditions, part of lactic acidosis is explained by metformin-induced impaired liver clearance.

## Conclusion

We described one of the largest series of patients with MALA and suggested a possible beneficial effect of dialysis in the care of this disorder. Larger and prospectively designed studies are clearly needed to draw firm recommendations on the treatment of MALA.

## Key messages

• MALA is of low (1%) prevalence in medical ICUs

• MALA is associated with a high (30%) mortality rate

• Prothrombin time on admission seems to be inversely related to survival

## Abbreviations

ICU: intensive care unit; MALA: metformin-associated lactic acidosis; SAPSII: simplified acute physiology score II.

## Competing interests

The authors declare that they have no competing interests.

## Authors' contributions

NP, AC, DB, LN, PEB and SG collected data. NP, NJ and SG analysed the data. NP and SG wrote the draft.
